# Biofilm vs. Planktonic Lifestyle: Consequences for Pesticide 2,4-D Metabolism by *Cupriavidus necator* JMP134

**DOI:** 10.3389/fmicb.2017.00904

**Published:** 2017-05-23

**Authors:** Thomas Z. Lerch, Claire Chenu, Marie F. Dignac, Enrique Barriuso, André Mariotti

**Affiliations:** ^1^UMR IEES-Paris, Institute of Ecology and Environmental Sciences of Paris, (Centre National de la Recherche Scientifique, UPMC, Institut National de la Recherche Agronomique, UPEC, IRD, Université Paris Diderot)Créteil, France; ^2^UMR ECOSYS, Écologie Fonctionnelle et Écotoxicologie des Agroécosystèmes (Institut National de la Recherche Agronomique, AgroParisTech, Université Paris-Saclay)Thiverval-Grignon, France

**Keywords:** biofilm, 2,4-D, *Cupriavidus necator* JMP134, PLFA-SIP

## Abstract

The development of bacterial biofilms in natural environments may alter important functions, such as pollutant bioremediation by modifying both the degraders' physiology and/or interactions within the matrix. The present study focuses on the influence of biofilm formation on the metabolism of a pesticide, 2,4-dichlorophenoxyacetic acid (2,4-D), by *Cupriavidus necator* JMP134. Pure cultures were established in a liquid medium with 2,4-D as a sole carbon source with or without sand grains for 10 days. Bacterial numbers and 2,4-D concentrations in solution were followed by spectrophotometry, the respiration rate by gas chromatography and the surface colonization by electron microscopy. In addition, isotopic techniques coupled with Fatty Acid Methyl Ester (FAME) profiling were used to determine possible metabolic changes. After only 3 days, approximately 80% of the cells were attached to the sand grains and microscopy images showed that the porous medium was totally clogged by the development of a biofilm. After 10 days, there was 25% less 2,4-D in the solution in samples with sand than in control samples. This difference was due to (1) a higher (+8%) mineralization of 2,4-D by sessile bacteria and (2) a retention (15%) of 2,4-D in the biofilm matrix. Besides, the amount of carbohydrates, presumably constituting the biofilm polysaccharides, increased by 63%. Compound-specific isotope analysis revealed that the FAME isotopic signature was less affected by the biofilm lifestyle than was the FAME composition. These results suggest that sessile bacteria differ more in their anabolism than in their catabolism compared to their planktonic counterparts. This study stresses the importance of considering interactions between microorganisms and their habitat when studying pollutant dynamics in porous media.

## Introduction

The functioning of microorganisms is closely linked to their distribution in a structured media, such as soil. A review by Kuzyakov and Blagodatskaya (2015) stressed the importance of considering the spatial and temporal heterogeneity of microbial processes in soils. They defined microbial hotspots as small soil volumes with much faster process rates and much more intensive interactions compared to the average soil conditions. Considering microbial hotspots is particularly important when studying the fate of pesticides in soils because of the very high spatial variability of their degradation. For example, Monard et al. (2012) showed that 2,4-D mineralisation variability was higher than that of a simple molecule, such as glucose, due to the spatial heterogeneity of specific 2,4-D degraders. Dechesne et al. (2014) reported that the presence and activity of pesticide degraders frequently displays non-random spatial patterns with coefficients of variation often exceeding 50%. Soil microorganism localization is indeed restricted to very small microhabitats comprising much <1% of total soil volume (Young et al., 2008) and covering <10^−6^% of the soil surface area (Young and Crawford, 2004). These habitats are composed of diverse microbial assemblages ranging from single colonies to biofilms (Hodge et al., 1998; Ekschmitt et al., 2005). *In situ* evidence of the role of such microhabitats on pesticide degradation is often difficult to obtain. Therefore, biofilms should be considered as relevant microbial hotspots for studying processes *in vitro*.

Biofilm research can be traced back to the 1930s when bacterial attachment and growth on solid supports was first noted and the term “surface effect” proposed (see Zobell and Anderson, 1936; Zobell, 1943). This effect describes the ability of solid surfaces to adsorb minute but demonstrable quantities of organic matter, thus retarding exocellular enzymes and hydrolysates diffusion away from surface-localized bacterial cells (Vanloosdrecht et al., 1990). Most of the batch studies on pesticide degradation have been done without taking into account this surface effect, using shaken-liquid medium, which favors bacteria that grow well in suspension. However, biofilms are considered to be a major mode of bacterial life in nature (Costerton et al., 1995). The use of liquid media might introduce a significant bias when studying pesticide degradation. Compared to their planktonic counterparts, sessile bacteria often benefit from physical and physiological interactions that enhance nutrient availability and, potentially, toxic metabolite removal (Davey and O'Toole, 2000). Cooperative and competitive cell–cell interactions that define biofilm form and function are influenced by the spatial arrangement of genotypes within a community (Nadell et al., 2016). The extracellular polysaccharide (EPS) matrix also provides a protection against a variety of environmental stresses, including pesticides exposure, which could explain the tolerance of microbial communities to such xenobiotics (Imfeld and Vuilleumier, 2012). While recent studies have demonstrated that biofilms induced changes in pesticide degradation rate, due to the modifications in the microbial communities (e.g., Verhagen et al., 2011; Tien et al., 2013), very few studies have focused on the consequences for the metabolism of pesticides of biofilm formation by a single strain of degrader. The first objective of the present study is to address this question.

The second objective of this study is to evaluate the potential change in membrane lipid profiles between planktonic and sessile bacteria. Indeed, phospholipid fatty acids (PLFA) are commonly used as biomarkers in soil microbial ecology. Such molecules provide information about the whole community structure of a sample, but also allow the targeting of microbial groups specifically involved in the degradation of a given pesticide when the latter has been labeled with ^13^C labeling. This method is called Stable Isotope Probing (SIP). PLFA were the first to be analyzed by SIP (Boschker et al., 1998) and then the technique was extended to nucleic acids (NA) a few years later (Radajewski et al., 2000). Although less taxonomically precise, lipid based method are much more sensitive than NA based SIP. This method has been successfully used to assess microbial community of 2,4-D degraders on long term incubation experiment (e.g., Lerch et al., 2009a) and even bound residues when the dilution of the labeling is high (Lerch et al., 2009b). In order to provide taxonomic information on the targeted communities, isotopically enriched lipid profiles have to be compared to existing fatty acid databases of cultured microorganisms. Thus, pure culture studies are still necessary not only to increase phylogenetic resolution of lipid biomarkers, but also to ensure their accuracy. Indeed, the fatty acid composition of the cell membrane is known to be affected by many stresses, such as high temperature (Heipieper et al., 1996), low pH (Baath and Anderson, 2003), heavy metals (Frostegard et al., 1993), organic pollutants (Rosas et al., 1980). More recently, the biofilm formation has also been showed to induce changes in membrane lipids (Benamara et al., 2011).

Here, we compared the biodegradation of 2,4-D by a pure strain of *Cupriavidus necator* JMP134 in the presence or absence of a solid phase. A previous study based on the same microbial model showed that *C. necator* JMP134 preferentially used C originating from the 2,4-D acetic chain for energy while C originating from the benzenic ring was rather used as C source (Lerch et al., 2007). In the present study, we hypothesize that adding sand grains to a culture will induce the development of a microbial biofilm that might change the bacterial metabolic activity and/or the retention of 2,4-D. We combined classical optical density measurements, respirometry, microscopy and isotopic analysis. The fatty acid composition of *C. necator* JMP134 has been reported to be modified by the nature of the growth substrate (Lerch et al., 2011). We expect that such potential change in fatty acid profiles and isotopic signature could also occur with the biofilm formation.

## Materials and methods

### Chemicals, culture, and growth conditions

Unlabelled 2,4-D (chemical purity > 99%, δ^13^C = −29.1‰) was purchased from Sigma-Aldrich Co., Ltd. and ring-U-labeled ^13^C-2,4-D (99% of chemical purity, isotopic enrichment > 98%) was obtained from Dislab'system (France). Before any experiments, the ^13^C-2,4-D level of enrichment was checked by GC-IRMS (see description below).

All cultures were grown in a minimum medium (MM) containing K_2_HPO_4_ (1.5 g.L^−1^), KH_2_PO_4_ (0.5 g L^−1^), (NH_4_)_2_SO_4_ (1 g.L^−1^), MgSO_4_ (H_2_O)_7_ (207 mg.L^−1^), ZnSO_4_ (H_2_O)_7_ (200 μg.L^−1^), MgCl_2_ (H_2_O)_4_ (10 μg.L^−1^), H_3_BO_3_ (5 μg.L^−1^), CoCl_2_ (H_2_O)_6_ (25 μg.L^−1^), CuSO_4_ (100 μg.L^−1^), NiCl_2_ (H_2_O)_6_ (5 μg.L^−1^), FeSO_4_ (H_2_O)_7_ (250 μg.L^−1^), and EDTA (ED4S) (125 μg.L^−1^). 2,4-D (250 mg.L^−1^) was added as the sole carbon source (the amount of C from EDTA was considered negligible compared to that in 2,4-D, 2,000 times higher). Two types of incubations were performed. Incubations with unlabelled 2,4-D were performed in Erlenmeyer's containing 200 mL of MM with or without (Control) 200 g of fine sterilized sand (sable de Fontainebleau, 100–200 μm, Prolabo). Incubations with ^13^C-labeled 2,4-D (δ^13^C = 134‰) were performed in serum bottles (120 mL) with Teflon® rubber stoppers crimped on with aluminum seals containing 50 mL of MM with or without (Control) 50 g of sand. There were 4 replicates per treatment in the unlabelled and ^13^C-labeled 2,4-D experiments. The sand was completely saturated and there was approximately 80 mL of free water phase. So, it was possible to shake and sample the liquid medium in the same way as for the control samples (without sand).

The inoculation of the pure *C. necator* JMP134 strain (courtesy of F. Martin-Laurent, INRA Dijon, France), collected at the end of the exponential growth phase, was done at a level of 10^5^ CFU.mL^−1^. Pre-cultures were performed in a TY liquid medium: 5 g.L^−1^ of tryptone (δ^13^C = −23.1‰), 3 g.L^−1^ of yeast extract (δ^13^C = −26.1‰) and 50 mg.L^−1^ of 2,4-D. According to the C content of each substrate, the average isotopic enrichment of the preculture was −23.9‰. The inoculum was rinsed once with phosphate buffer to limit the addition of C from the TY medium in the MM. All batch cultures were performed at 25°C in the dark on a rotary shaker at 150 rpm.

### Bacterial and 2,4-D concentration measurements

For all experiments (unlabelled and ^13^C-labeled 2,4-D experiments) we estimated 2,4-D (% of initial amount) and bacterial concentrations [Log(CFU).mL^−1^] after 1, 2, 3, 5, and 10 days of incubation by measuring the optical density in the liquid medium using a Lambda 5 (Perkin-Elmer) spectrophotometer (λ_2,4−*D*_ = 282 nm and λ_Bacteria_ = 600 nm). At each sampling date, 4 replicates of each treatment were destructively sampled for microscopy and carbohydrates measurements (unlabeled 2,4-D experiments) or for Fatty Acid Methyl Ester (FAME) profiling (^13^C-labeled 2,4-D experiments).

### Scanning electron microscopy

Scanning Electron Microscopy (SEM) of sand surface was performed on the unlabelled 2,4-D experiments at each sampling date. In order to preserve the original structure of the sample we used a SEM equipped with a low temperature system (Chenu and Tessier, 1995). Sand samples of about 1 mm^3^ were mounted on the metal stubs of a specimen holder (Hexland cryostrans system CT 1,500) and were cryofixed by immersion in a nitrogen slush at −200°C, transferred to the microscope preservation chamber at −180°C where they were fractured with a cold blade. Ice was partially sublimated under vacuum at about −80°C for 20 min, and the disappearance of ice in the pores of the samples was controlled by observation under the microscope. The surface of the sample was coated at −180°C with gold, and the samples were then introduced in the refrigerated columns of the microscope (Philips SM 525 SEM) to be observed at a temperature < −160°C. For each case date of sampling three replicates were observed.

### CO_2_ measurements

Measurements of the amount and the ^13^C enrichment of the CO_2_ evolved were performed during the ^13^C-labeled 2,4-D experiments. At each sampling date, 3 gas samples were taken from the serum bottle headspace and the CO_2_ was directly quantified with a micro-GC (Agilent 3,000A) equipped with a HP-Plot-U column with helium as the carrier gas. The gas sample was introduced into the micro GC via a built-in vacuum pump. The column and the detector were maintained at 80°C. The isotopic content of the CO_2_ was measured with a GC-IRMS (Isochrom Optima, Micromass) on 3 other gas samples injected with a syringe into the GC. The GC was equipped with a Haysep Q column (Valco Instruments Co. Inc.) and helium as the carrier gas. The injector and detector were maintained at 180°C. The column temperature was programmed at 80°C then ramped at 30°C per min to 160°C. The δ^13^C standard deviations were 0.1‰or better. At the beginning of the experiment and after measuring CO_2_, all the flasks were flushed with reconstituted air (19% O_2_, 81% N_2_).

### Carbohydrate analysis

Carbohydrate concentrations of each sample were measured as hexoses by the phenol-sulfuric method as previously described by Dubois et al. (1956). Briefly, 2.5 μl of phenol (80%), and 250 μl of concentrated sulfuric acid were added to 100 μl of each cell wall fraction. Following incubation at 30°C for 30 min, color was detected by an automated plate reader at 490 nm. The carbohydrate concentration was determined using glucose controls (37.5, 75, 150, and 300 g.mL^−1^). Three analytical replicates were performed for each experimental replicate and each date of sampling.

### Lipid extraction and analysis

At each sampling date, cells from control samples were harvested by centrifugation (3,500 g for 20 min at 4°C) using a Beckman-Coulter Adventi J20 XP1 and then lyophilised before lipid extraction (see protocols described below). Samples containing sand were first filtered (GF/F, < 0.7 μm, Whatmann) and then lyophilised with the filter. For ^13^C-labeled 2,4-D experiments, the 4 replicates samples were put together to obtain a sufficient amount of biomass. The total lipid fraction was preferred to the PLFA method because it may also contain information about 2,4-D and its metabolites, as these are extractable in the FAME extractants (Lerch et al., 2007). Briefly, samples were extracted with a mixture of dichloromethane and methanol by using the modified Bligh/Dyer method as described previously by White et al. (1979). The total lipids obtained were transesterified with BF_3_/Methanol to recover the FAME (Morrison and Smith, 1964) and 2,4-D as methyl-2,4-D (Cochrane et al., 1981) in hexane/ether.

The FAME were separated and quantified by GC with flame ionization detection (GC-FID), identified by mass spectrometry (GC-MS) and their individual isotopic analysis was done by isotopic ratio mass spectrometry (GC-IRMS) as follows. The fatty acid methyl esters were analyzed by capillary GC-FID on a Hewlett-Packard model 5890 series II chromatograph with a 60 m SGE BPX5 column (0.32 mm inside diameter and 0.25 μm film thickness) with helium as the carrier gas. The injector and detector were maintained at 280°C. The column temperature was programmed at 50°C for 1 min, then ramped at 5°C per min to 300°C, followed by an isothermal period of 10 min. Samples were injected in splitless mode. The preliminary peak identification was done by comparison of retention times with commercial standards for FAME (BAME Mix, Supelco) and some molecules, such as 2,4-D, 2,4-DCP (2,4-dichlorophenol) and 3,5-CC (3,5-dichlorocatechol). Results were expressed as equivalent peak responses to these standards. Peak identification was completed by GC-MS of selected samples with a Hewlett-Packard model 6890N gas chromatograph interfaced to a HP 5373 quadrupole mass spectrometer by using the same column and temperature program as previously described. The electron impact ion source was maintained at 250°C, and the quadrupole temperature was 150°C. Mass spectra were obtained by electron impact at 70 eV.

For ^13^C-labeled 2,4-D experiments, the individual δ^13^C measurement of each FAME was performed in triplicate on an Isochrom III Isotopic mass spectrometer (Micromass-GVI Optima) coupled to a GC HP5890 with the same column and conditions as those previously described for GC-MS analysis. The column effluent was combusted on-line in an oxidation oven (copper-nickel-platinum catalyst at 980°C) and passed through a reactor with elemental copper (600°C) to reducing NO*x* and remove surplus O_2_. Combustion gases were purified in a cryogenic trap containing liquid N_2_ (−100°C).

### Isotopic calculations

The standard notation for expressing high-precision gas IRMS results in δ is defined as follows:

(1) $$\begin{array}{r} \delta^{\text{13}}C\left(‰\right)=\left(\frac{R_{Sample}}{\text{1000}}+\text{1}\right)\times R_{VPDB} \end{array}$$

where *R*_*Sample*_ and *R*_*VPDB*_ are the ^13^C/^12^C isotope ratios corresponding respectively to the sample and to the international internal standard Vienna PeeDee Belemnite (*R*_*VPDB*_ = 0.0112372 ± 0.0000090).

The derivatization of the fatty acids introduces one additional carbon which is not present in the parent compound and which alters the original isotope ratio of the fatty acids. However, the derivatization process introduces a distinct reproducible fraction that is constant for each fatty acid (Goodman and Brenna, 1992). The measured isotope ratios of the FAME were corrected for the isotope ratio of the methyl moiety to obtain the isotope ratios of the fatty acids. This was done by using the Formula (2):

(2) $$\begin{array}{r} \delta^{\text{13}}C_{FA}=\frac{\left(Cn+\text{1}\right)\times \delta^{\text{13}}C_{FAME}-\delta^{\text{13}}C_{MetOH}}{Cn} \end{array}$$

where δ^13^C_*FA*_ is the δ^13^C of the fatty acid, C_*n*_ is the number of carbons in the fatty acid, δ^13^C_*FAME*_ is the δ^13^C of the FAME, and δ^13^C_*MeOH*_ is the δ^13^C of the methanol used for the methylating reaction (−63.2‰) to calculate the isotope ratios of the fatty acids.

Considering the 2,4-D as the sole carbon source in the medium, we used the mass conservation Equation (3) and the mixing isotopic mass Equation (4) in order to calculate the amount of ring-C and chain-C in a considered fraction:

(3) $$\begin{array}{r} {C\;}_{Fraction}={C\;}_{Ring}+{C\;}_{Chain} \end{array}$$

(4) $$\begin{array}{r} \delta^{\text{13}}{C\;}_{Fraction}\;\times {C\;}_{Fraction}=\;\delta^{\text{13}}{C\;}_{Ring}\times {C\;}_{Ring}+\;\delta^{\text{13}}{C\;}_{Chain}\\ \;\times {C\;}_{Chain} \end{array}$$

where C_*Fraction*_ is the amount of total C, C_*Ring*_ is the amount of C coming from the 2,4-D benzenic ring (ring-C) and C_*Chain*_ is the amount of C coming from the 2,4-D acetate chain (chain-C). By simplifying equations (3) and (4), the equations giving C_*Ring*_ and C_*Chain*_ in a given fraction are the following:

(5) $$\begin{array}{r} {C\;}_{Chain}=\;{C\;}_{Fraction}\times \left(\frac{\delta^{\text{13}}{C\;}_{Fraction}-\;\delta^{\text{13}}{C\;}_{Ring}}{\delta^{\text{13}}{C\;}_{Chain}-\;\delta^{\text{13}}{C\;}_{Ring}}\right) \end{array}$$

(6) $$\begin{array}{r} {C\;}_{Ring}=\;{C\;}_{Fraction}\times \left(\frac{\delta^{\text{13}}{C\;}_{Fraction}-\;\delta^{\text{13}}{C\;}_{Chain}}{\delta^{\text{13}}{C\;}_{Ring}-\;\delta^{\text{13}}{C\;}_{Chain}}\right) \end{array}$$

where δ^13^C_*Fraction*_ is the measured value, δ^13^C_*Chain*_ (−32.1‰) and δ^13^C_*Ring*_ (+177‰) are the isotopic signatures of the acetate chain and benzene ring previously determined by Lerch et al. (2007). Equations 5 and 6 were used for every FAME analyzed and the CO_2_ evolved. It is important to notice that the C of the inoculum was neglected since it was at least 100 fold lower than the amount of biomass C after 1 day of incubation.

### Statistical analysis

Significant differences among treatments (with or without sand) were tested by ANOVA followed by Tukey's HSD test. Principal component analysis (PCA) was performed both on total FAME profiles and on the percentage of C_*Ring*_ incorporated into each FAME. All multivariate analyses were performed using the “ade4TkGUI” package in R while graphical representations were performed with SigmaPlot 11.0. All statistical analyses were performed with R software (version 2.12.0 R Development Core Team, 2008).

## Results

### *Cupriavidus necator* JMP134 biofilm formation

**Figure 1:**
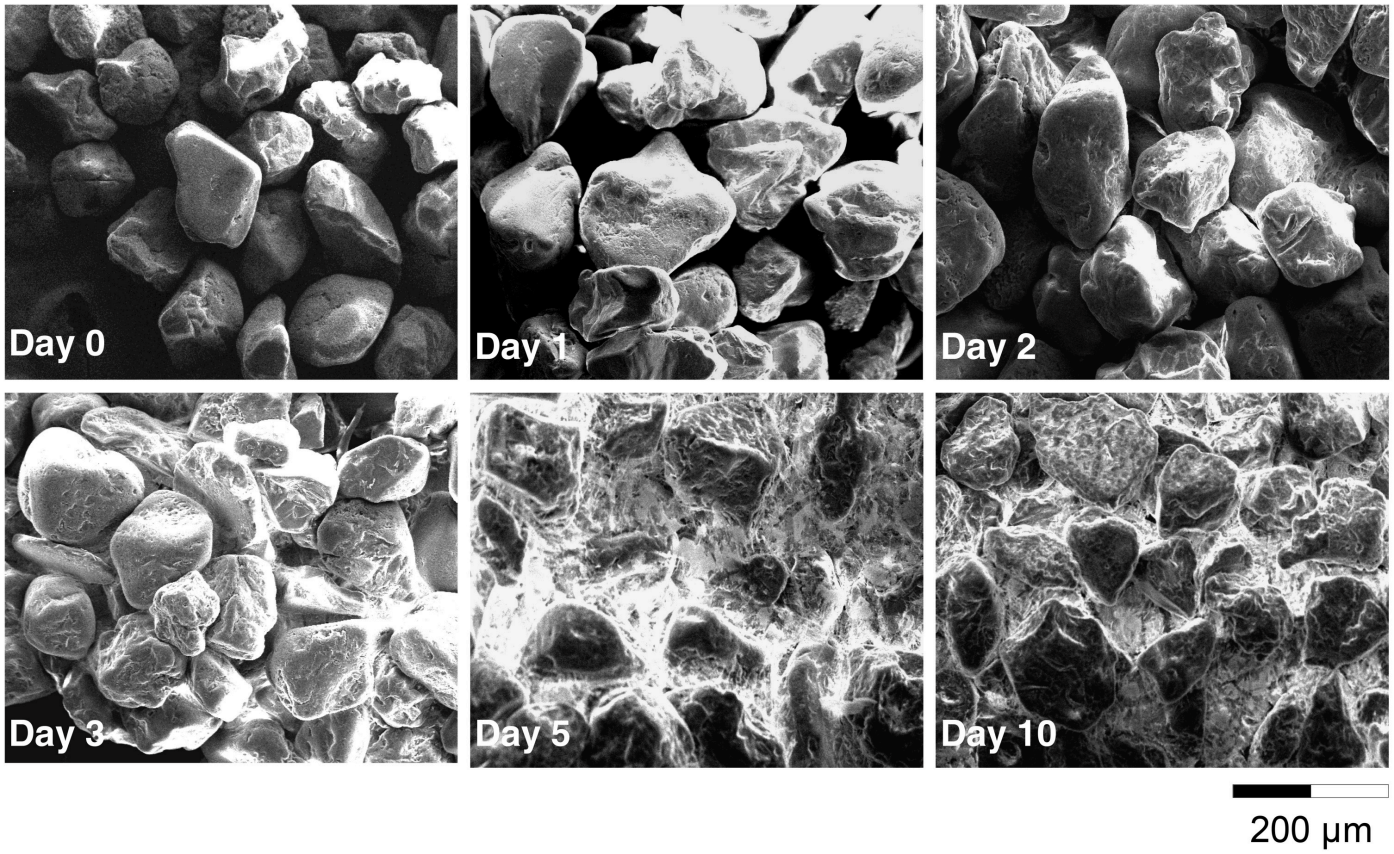
**Scanning Electron Microscopy of the sand grains surface after 0, 1, 2, 3, 5, and 10 days of incubation**.

Electron microscopy observations revealed that *C. necator* JMP134 was able to form a biofilm in a minimum medium containing only 2,4-D as C source and sand as a solid phase. After just 1 day of incubation, bacteria (about 1 μm diameter) and exopolymers (thin white filament) were detected on the surface (Figure S1). After 3 days, the density of sessile bacteria increased slightly as well as the amount of polymers between sand grains. After 5 days, sand grains were totally recovered by a film of polymers of approximately 1 μm thick. When analyzing sand grains at a larger scale (Figure 1), we observed that the porous media became totally clogged by biopolymers after 5 days of incubation.

### 2,4-D biodegradation

**Figure 2:**
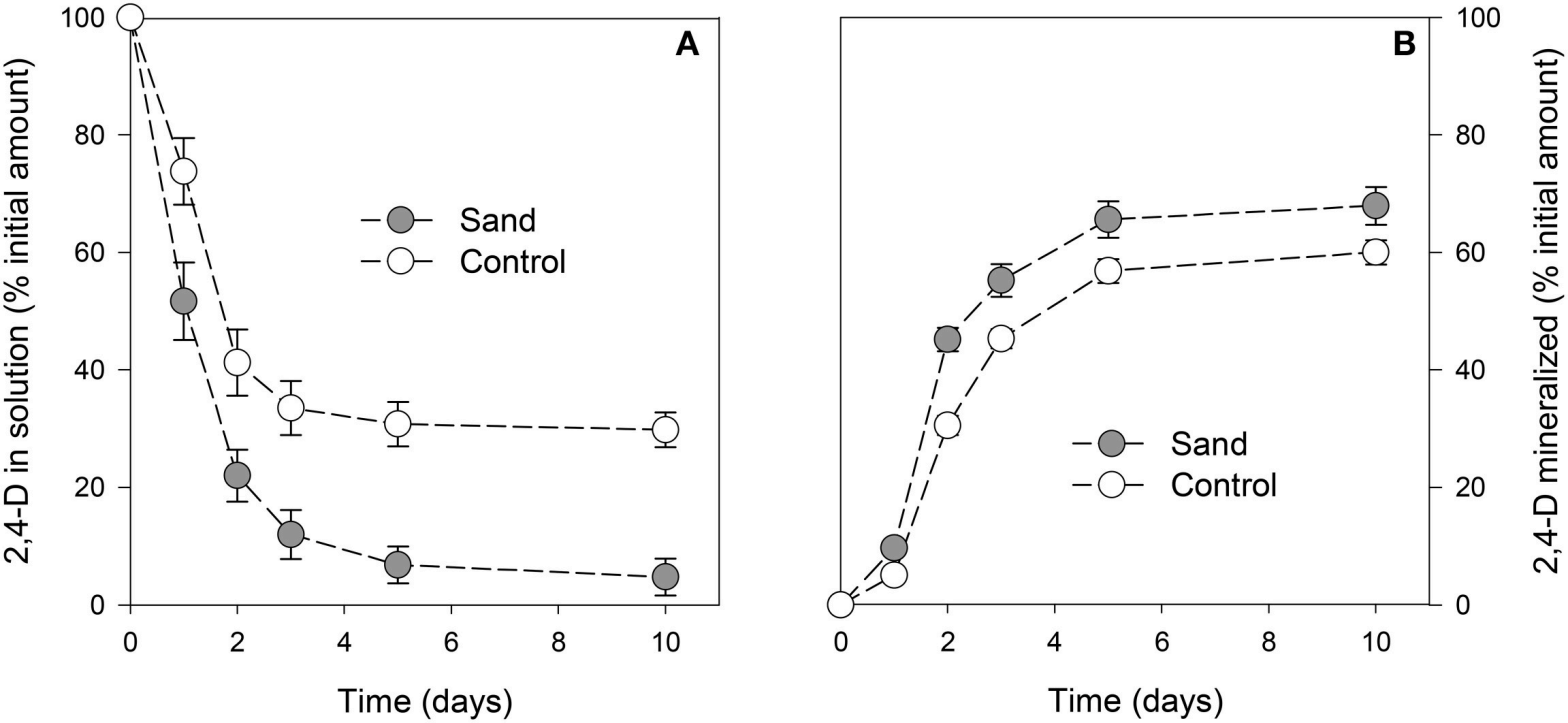
**Proportion of 2,4-D in solution (A)** or mineralized **(B)** with time. The gray circles and white circles represent incubation with or without addition of sand, respectively. Error bars correspond to the standard deviation calculated for 3 replicates.

The concentration of 2,4-D in solution was always (*P* < 0.001) lower in samples containing sand than in control samples (Figure 2A). After 1 day of incubation, the amount of 2,4-D in samples with sand was already significantly lower than for control samples. For both curves, a minimum was reached after 3 days of incubation. At the end of the experiment, the 2,4-D concentrations in solution were 5 ± 3% and 30 ± 3% of the initial amount in samples with or without sand, respectively. The amount of 2,4-D mineralized throughout the experiment was always higher (*P* < 0.001) in samples containing sand (Figure 2B). After 10 days of incubation, the cumulative amounts of CO_2_ evolved were 68 ± 3% and 60 ± 2% of the initial amount of C in the medium in samples with or without sand, respectively.

It should be mentioned that whatever the sampling date, the lipid fraction of control samples did not contain any residues of 2,4-D or its metabolites (data not shown). In contrast, the analysis of the lipid fraction extracted from samples containing sand revealed the presence of 2,4-D (Figure S2). Other 2,4-D metabolites were not detected. The quantity of 2,4-D in this fraction corresponded to 9 ± 3% of the initial amount on the first day and then remained stable between 13 ± 4 and 15 ± 4% of the initial amount from day 2 onwards.

### Amount of microbial biomass, fatty acids, and carbohydrates

**Figure 3:**
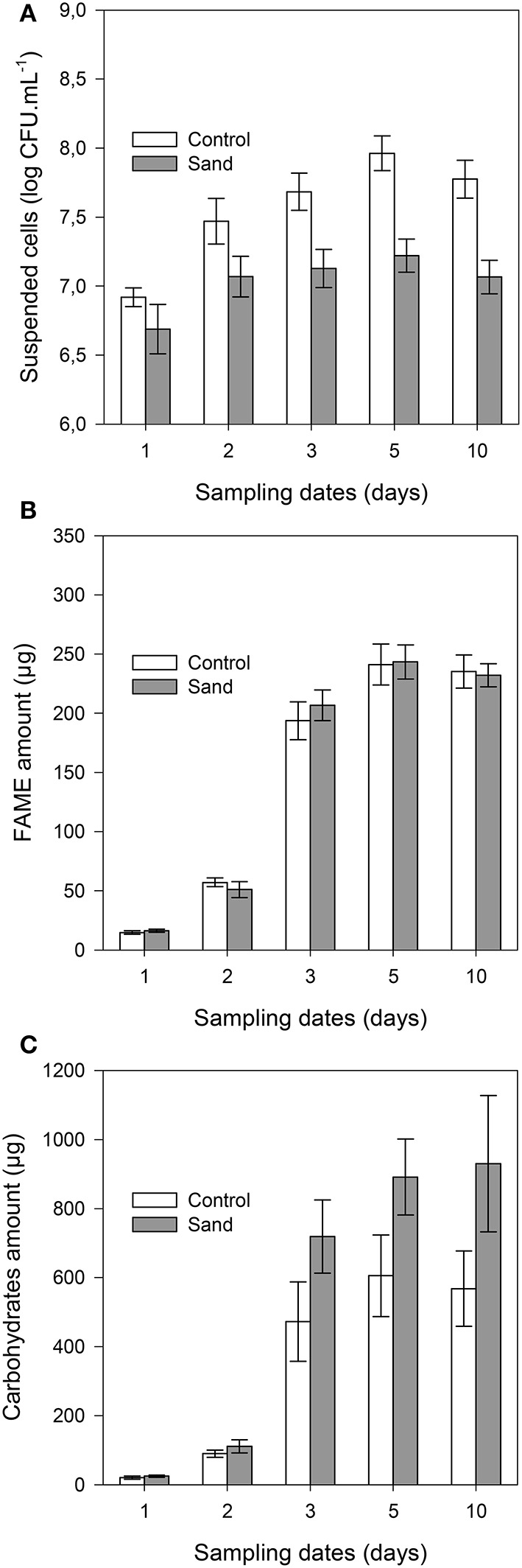
**Cell concentration in solution (A)**, total amounts of FAME **(B)**, and carbohydrates **(C)** per sample with time. The gray bars and white bars represent incubation with or without addition of sand, respectively. Error bars correspond to the standard deviation calculated for 3 replicates.

The concentration of suspended cells in solution was always (*P* < 0.001) lower in samples containing sand than in control samples (Figure 3A). The planktonic bacterial concentration reached a plateau after 3 days for control (approximately 9 × 10^7^ cells.mL^−1^) and 2 days for sand samples (2 × 10^7^ cells.mL^−1^). It should be noted that no significant differences were observed between unlabelled and ^13^C-labeled experiments, indicating that both incubations were similar. The total amount of FAME (detailed below) was similar in both treatments throughout the incubation (Figure 3B), indicating similar microbial biomass in planktonic and biofilm cultures. The maximum amount of FAME was reached after 5 days of incubation with 243 ± 14 μg and 241 ± 17 μg per replicate for sample with or without sand, respectively. The amount of total carbohydrates (Figure 3C) was also significantly (*P* < 0.001) higher in the biofilm culture than in the suspended cell cultures from 3 to 10 days of incubation (approximately 47 to 63% more).

### Fatty acid composition

**Figure 4:**
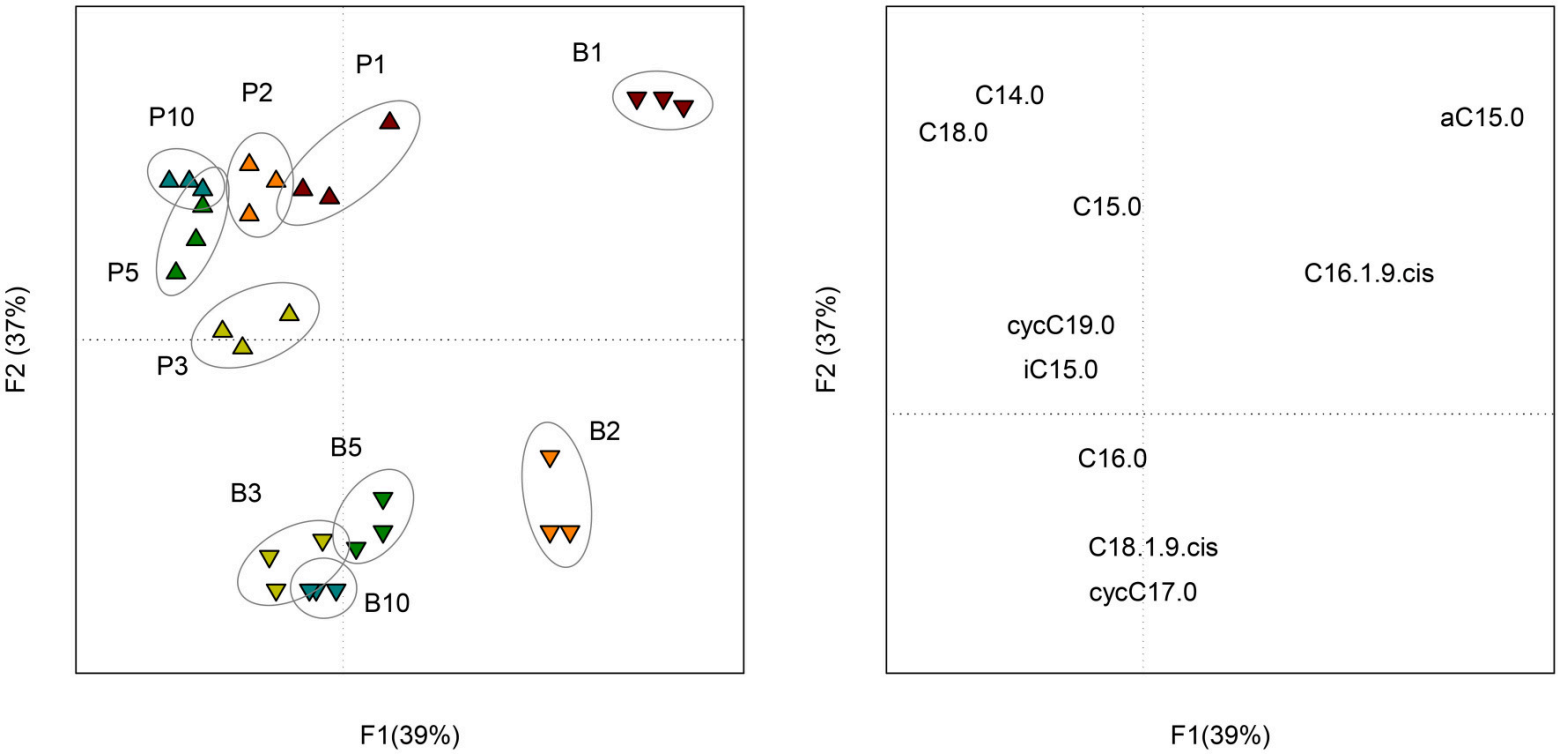
**Scores (left) of the PCA performed on the composition (molar percentage) of FAME profiles of planktonic (P) and biofilm (B) cells at each sampling date (1, 2, 3, 5, and 10 days of incubation)**. Loadings (right) of the PCA representing the 10 FAME detected in the profiles.

The 10 most concentrated FAME identified by GC analyses are expressed in percentage of their relative amount (Figure S3). Five other FAME (iC16:0, iC17:0, C17:0, C18:0, C18:1ω9t and C19:0) were present at trace levels (relative amount < 0.5%) and were not represented. The ANOVA revealed significant differences among sampling dates, but, no significant differences between sessile bacteria (control) and sessile + planktonic bacteria (+ sand), except at day 10. At this sampling date, the relative amount of C16:0 was significantly lower and that of the cycC17:0 was significantly higher for samples containing sand grains. In both treatments, four major FAME (C16:1ω9c, C16:0, cycC17:0, and C18:1ω9c) accounted for approximately 90% of total FAME. Six minor fatty acids (C14:0, iC5:0, aC15:0, C15:0, C18:0, and cycC19:0) were found at a level of approximately 10%. The proportion of three of the major FAME (C16:1ω9c, cycC17:0, and C18:1ω9c) clearly evolved with time (*P* < 0.001). The evolution of C16:0 proportion was less significant (*P* < 0.05). It should be mentioned that the cyclopropyl/precursor ratio (cycC17:0+cycC19:0/C16:1ω9c + C18:1ω9c) increased significantly (*P* < 0.001) for both treatments (Table S1). This ratio was significantly higher (*P* < 0.01) for sessile bacteria, especially from day 3 onwards. PCA performed on FAME profiles (Figure 4) showed change with time (*F1* = 39%) and a distinction between planktonic and sessile cells (*F2* = 37%). A redundancy analysis showed that 95% of the variability was explained by these two factors.

### Isotopic analysis

The isotopic analysis of the CO_2_ did not show significant differences in the origin of C respired between samples with or without sand (Figure S4). In both treatments, the proportion of C originating from the benzenic ring (C_*Ring*_) was slightly lower in the CO_2_ (≈ 73%) than in the 2,4-D (75%), indicating that *C. necator* JMP134 preferentially used the C originating from the acetic chain (C_*Chain*_) for energy.

**Figure 5:**
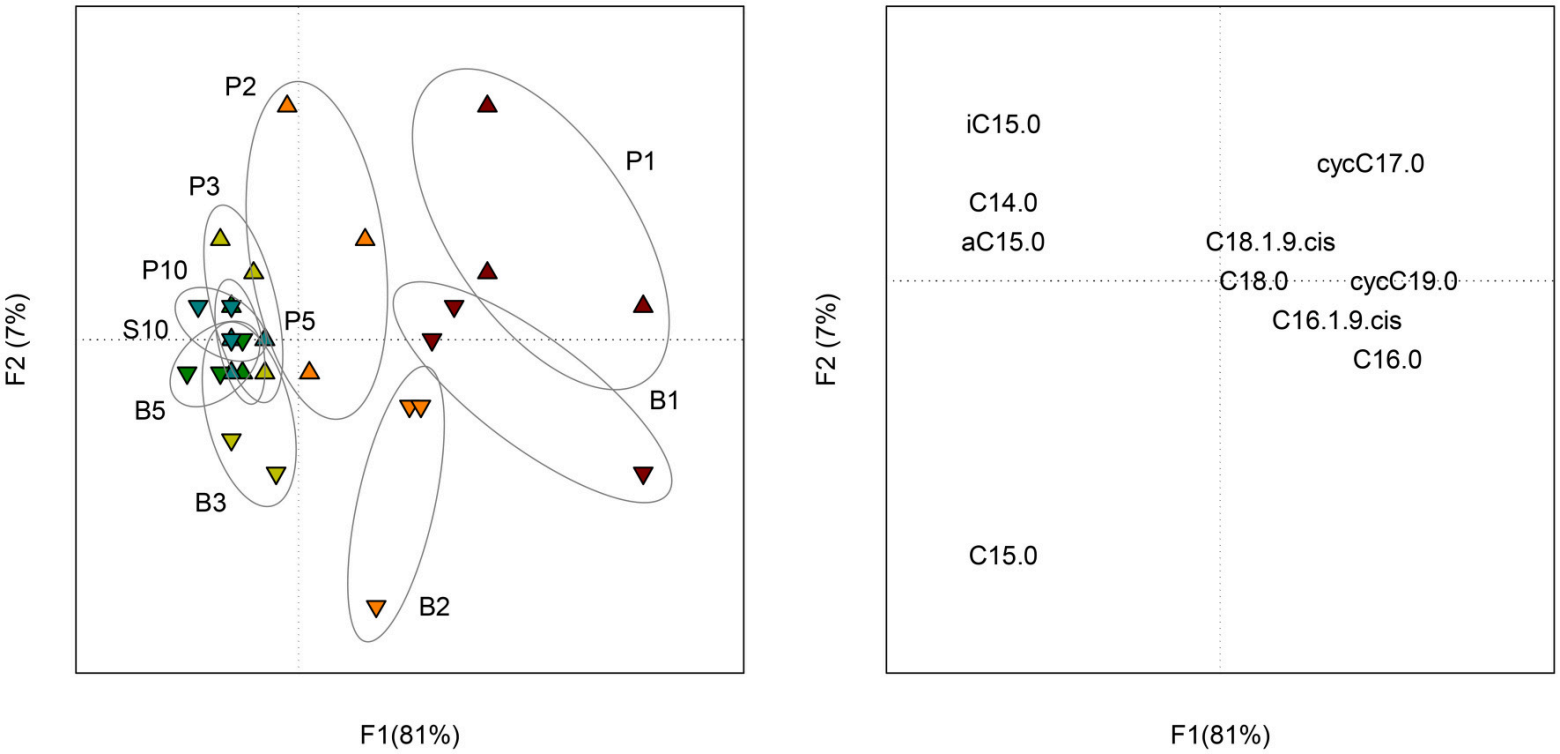
**Scores (left) of the PCA performed on the proportion of C_***Ring***_ in each FAME of planktonic (P) and biofilm (B) cells at each sampling date (1, 2, 3, 5 and 10 days of incubation)**. Loadings (right) of the PCA representing the 10 FAME detected in the profiles.

The isotopic analysis of individual FAME did not reveal significant differences between samples containing sand and control samples. As a result, the proportion of C originating from the benzenic ring (C_*Ring*_) and the acetic chain (C_*Chain*_) were similarly incorporated in sessile and planktonic bacteria. For both treatments, the proportion of C_*Ring*_ in FAME increased with time (Figure S5). PCA performed on the proportion of C_*Ring*_ in FAME (Figure 5) showed a small change with time (*F1* = 81%) and a little distinction between planktonic and sessile cells during the first 2 days of incubation (*F2* = 7%).

## Discussion

### Biofilm development of *C. necator* JMP134

The similarity of the total FAME amounts in both treatments (with or without sand) suggested that there was no difference in the total microbial biomass. Spectrophotometric measurements of the bacterial density in the liquid phase showed that *C. necator* JMP134 cultures in the presence of 2,4-D presented similar growth as reported previously (Lerch et al., 2007, 2011). For both treatments, the exponential phase reached its maximum after 3 days followed by a stationary phase that lasted for the next 2 days. Thereafter, there was a similar stage of cellular death with decreasing OD. While both treatments showed the same kinetics, the concentration of suspended bacteria in samples containing sand was approximately 25% that of control samples. In parallel, electron microscopy observations showed that bacterial cells were already attached to the sand grain surfaces after 1 day of incubation. Thus, we conclude that from the early growth stage, most of the cells were attached to the sand grains than being in suspension. By using mass conversion of cell density and with comparison to optical density measurements, we estimated that approximately 80% of the cells in samples containing sand were fixed to the mineral phase from 2 days onwards. Assuming sand grain were perfect spheres (specific area of 0.02 m^2^.g^−1^ and density of 2.7 g.cm^−3^), the surface density of sessile bacteria was estimated to be 0.02 to 0.1 cell.μm^−2^, which is consistent with SEM analyses.

Most biotreatment experiments using biofilms are enhanced by previous encapsulation and/or support material with high surface area in conjunction with co-substrate to enhance the immobilization of the cells (e.g., Yanez-Ocampo et al., 2009). Our study showed that *C. necator* JMP134 is capable to rapidly attach to sand grains to reach a density of 0.1 cell per μm^2^ and develop a thin biofilm using 2,4-D as a sole carbon source of a biofilm with the cell. The ability of this bacteria to form biofilm has been reported previously in the literature. *Alcaligenes eutrophus* AE1308, a transconjugant of *A. eutrophus* CH34 and *A. eutrophus* JMP134 (former name of *C. necator* JMP134) have been successfully used to form active biofilm into membrane reactor for wastewater treatment (Diels et al., 1995). Other studies carried out in different environments showed that strains similar to *C. necator* JMP134 were able to form biofilms: *Ralstonia eutropha* (Steinle et al., 1998; Simkus et al., 2004), *Ralstonia solanacaerum* (Tans-Kersten et al., 2001; Kang et al., 2002), *A. eutrophus* (Mergeay et al., 1985; VanRoy et al., 1997), *Alcaligenes denitrificans* (Elvers et al., 1998), *Alcaligenes xylosoxydans* (Meade et al., 2001). We did not measure exactly the surface area and thickness of the biofilm. However, electron microscopy images showed that after 3 days of incubation, the thickness of the biofilm was approximately that of the bacteria (c.a. 1 μm), while the surface area of sand grains was almost entirely covered with polymers. Karthikeyan et al. (1999) reported that the thickness/area ratio of a biofilm was correlated to physicochemical characteristics of the organic pollutants being degraded by the biofilm, such as the octanol/water partition coefficient (*K*_*OW*_). It is hypothesized that a close positioning of cells is a disadvantage when cells compete for a labile carbon source. The more recalcitrant and hydrophobic is a molecule, the higher thickness/surface area ratio of the biofilm. This finding is in line with the observation we did with 2,4-D, a very soluble molecule: the biofilm surrounding cells was relatively thin but covered a large part of the grain sand surface. We also observed that after 5 days, all pores were clogged, which certainly modified the diffusion of substrates and metabolites (Chenu and Roberson, 1996) as well as exchanges between the liquid and the solid phases.

### Influence of biofilm on biodegradation

Cell immobilization has been employed for biological removal of pesticides because it allows maintaining a catalytic activity over long periods of time. Advantages over cultures using suspended cells include greater cellular content in the support, enhanced cellular viability (weeks or months) and greater tolerance to high concentrations of pollutants. For example, a membrane reactor composed of a microbial consortium showed a better removal of 2,4-D than activated sludge (Gonzalez et al., 2006). Our results suggest that even a single population of bacteria is able to remove more 2,4-D when organized into a biofilm. Some studies using bacterial strains similar to *C. necator* JMP134 showed that the cell immobilization enhanced the removal of monochlorophenol (Balfanz and Rehm, 1991; Menke and Rehm, 1992) or dichlorophenol (Steinle et al., 1998). Here, we showed that the greater 2,4-D decrease in the liquid medium was partly due to a higher mineralisation. It could be hypothesized that metabolic activity of the cell is higher when immobilized than in planktonic form (Kinniment and Wimpenny, 1992). In the present study, the total amount of biomass was not affected by the bacterial attachment onto the surface. However, SEM observations and carbohydrate measurements suggested that higher exopolymer amounts where produced when *C. necator* JMP134 was grown with sand. This EPS production likely required more energy, which could also explain the relative increase in 2,4-D mineralisation. The better efficiency of 2,4-D uptake may be related to the exchange of metabolites between cells and/or the nutrient recycling after cell death (Laspidou and Rittmann, 2002, 2004). These possible advantages of sessile bacteria over their planktonic counterparts may explain results obtained in this study and also those previously published (Wolfaardt et al., 1998; Karthikeyan et al., 1999).

### Influence of biofilm on 2,4-D retention

While the additional 2,4-D removal from the liquid medium was approximately 30% of added substrate in samples containing sand, only 10% more of added 2,4-D was mineralized. 2,4-D directly from the immobilized cells represented approximately 15% of the added substrate after 2 days of incubation (data not shown), which could explain the difference between the 2 treatments. Since 2,4-D is not adsorbed on the sand grains (preliminary experiments, data not shown) and the accumulation of 2,4-D into the cells is unlikely to happen (Lerch et al., 2007), we hypothesize that the retention of the pesticide was mainly due to the exopolysaccharides (EPS) matrix. It seems unlikely that EPS surrounding directly the immobilized cells may play a major role with regard to the low thickness (1 μm) of the biofilm in this area. However, SEM images revealed much more EPS in the porosity of the sand grains. In these specific area, the 2,4-D diffusion coefficient in the EPS matrix could be lower than in water, as shown already for glucose (Chenu and Roberson, 1996). Many authors have considered this phenomenon as a microbial process that enables to trap nutrients in various environments (Decho, 1990). The EPS matrix may also adsorb 2,4-D in soils as it has been reported for other molecules, such as dissolved organic matter (Sherr, 1988; Kaiser and Guggenberger, 2003), and organic pollutants (Flemming, 1995; Holden et al., 1997; Spath et al., 1998; Holden et al., 2002). An accumulation of pesticide diclofop in the EPS matrix was reported by Wolfaardt et al. (1994) with no consequence on its mineralisation rate. In our study, the accumulation of 2,4-D in the biofilm was accompanied by a higher mineralisation rate and higher carbohydrate amounts that are likely involved in EPS production. Considering that retention within the EPS matrix can allow further chemical transformation that would not happen in suspension (Confer and Logan, 1998), the increase in carbohydrate production could be a storage strategy to improve microbial survival since the production of biopolymers changed the habitat locally and improve substrate immobilization. In soils, the retention of 2,4-D on such biopolymers might play an important role in the formation of non-extractable residues (Lerch et al., 2009a) but also on their mineralisation (Lerch et al., 2009b).

### Influence of biofilm on PLFA profiles

The ANOVA performed on each FAME did not reveal significant differences between sessile and planktonic bacteria. However, the second principal component (37% of the variability) of the PCA performed on the whole FAME profiles was partly linked to the microbial lifestyle. When comparing the cyclopropyl/precursor ratios, we observed that sessile bacteria synthetized more cyclopropyl fatty acid than planktonic bacteria after 3 days of incubation. For both treatments, important changes in FAME profile were related to the sampling date (39% of the variability). These results suggest that the *C. necator* JMP134 FAME composition was influenced by the growth stage as much as by its attachment to a solid surface. Such growth stage influence on FAME composition is important to consider when interpreting FAME (or PLFA) profiles in complex environments. Variations in the lipid biomarker composition are not only due to the modification of the microbial composition but also to physiological changes. For example, the sensitivity of FAME profiles to change in growth substrate has been reported (Lerch et al., 2011). According to the present study, the bacterial sessile mode of life also alters the signature of lipid biomarkers.

It should be noted that we did not analyse hydroxy-fatty acids in this study since a sylilation is a prerequisite for GC analyses and this derivatisation involves the incorporation of many exogenous C atoms with their own isotopic signature. These lipopolysaccharides (LPS) components have been reported in the FAME profile of *R. eutropha* B8562, a strain similar to *C. necator* JMP134 (Volova et al., 2006). The LPS amount is likely to increase with the formation of biofilm (Brandenburg et al., 2003; Keinänen et al., 2003). Thus, it could be hypothesized that the proportion of hydroxy-fatty acids also increases during this phenomenon. However, most of the studies using lipid biomarkers for *in situ* assessment of the microbial communities do not consider hydroxy-fatty acids.

The analysis of FAME isotopic signatures gives a good estimate of the whole biomass isotopic signature (Lerch et al., 2007, 2009a). Here, we did not report significant changes in the FAME ^13^C enrichment between sessile and planktonic bacteria, suggesting that the formation of a biofilm by *C. necator* JMP134 did not change its catabolic pathway. As observed for the FAME profiles, the main changes in the isotopic signature were due to the growth stage. Consequently, the formation of biofilms in complex environments may not alter the isotopic signature of a mono-specific population.

## Conclusion

This study first reports that the *C. necator* JMP134 is able to very rapidly form a biofilm in a minimum medium supplemented with 2,4-D as a sole carbon and energy source. This ability induced direct consequences both on the mineralization and the retention of 2,4-D, leading to a better removal (25%) of the molecule from the solution. The accumulation of 2,4-D by EPS may be an active mechanism which enables *C. necator* JMP134 to use this molecule afterwards (e.g., when environmental conditions are more favorable). Considering the importance of the EPS matrix in this possible storage strategy, the increase in carbohydrates that constitute this biopolymer may be considered as an investment of energy and carbon to change locally the microbial habitat. This phenomenon may play an important role in the attenuation of pesticides in soils and other porous media since this kind of molecule is added intermittently in the microbial ecosystem. Isotopic analysis revealed that no major change of 2,4-D metabolism occurred when bacteria formed a biofilm: *C. necator* JMP134 preferentially used C originating from the acetic chain for energy while C originating from the benzenic ring is rather used as C source. The individual molecular analysis confirmed that the origin of 2,4-D carbon used for the synthesis of fatty acids did not differ between planktonic and sessile cells. However, we observed that the formation of the biofilm partially changed FAME profiles, which should be considered when using the signature of lipid biomarkers for *in situ* analysis. This study showed that physiological changes subsequent to biofilm formation mainly involved anabolic and not catabolic pathways.

## Author contributions

TL conceived the study, performed analyses and wrote the article. CC, MD, EB, and AM directed research and contributed substantially to the writing of the manuscript.

### Conflict of interest statement

The authors declare that the research was conducted in the absence of any commercial or financial relationships that could be construed as a potential conflict of interest. The handling Editor declared a shared affiliation, though no other collaboration, with the authors and states that the process nevertheless met the standards of a fair and objective review.

## Supplementary material

Figure S1 **Scanning Electron Microscopy of the sand grains surface at 0, 1, 2, and 3 days of incubation**.

Figure S2 **Example of chromatograms (GC-FID) of the lipid fraction obtained from the sand samples at each sampling date. 2,4-D but no metabolites were detected**. The other peaks corresponded to the bacterial fatty acids.

Figure S3 **Composition (molar percentage) of ***C.necator*** JMP134 FAME profiles in the control (upper) and sand (lower) samples at each sampling date**. Error bars correspond to the standard deviation calculated for 3 replicates.

Figure S4 **Proportion of C_***Ring***_ in the CO_**2**_ evolved during the 10 day incubation of ***C.necator*** JMP134 in the control (white circles) and sand (gray circles) microcosms**. Error bars correspond to the standard deviation calculated for 3 replicates. Dash line represents the proportion of C_*Ring*_ in the substrate (2,4-D) used.

Figure S5 **Proportion of C_***Ring***_ in each FAME of ***C.necator*** JMP134 for the control (A)** and sand **(B)** microcosms at each sampling date. Error bars correspond to the standard deviation calculated for 3 replicates. The gray line represents the proportion of C_*Ring*_ in the substrate (2,4-D) used.

Table S1 **Evolution of the cyclopropyl/precursor ratio (cycC17:0 + cycC19:0/C16:1ω9c + C18:1ω9c) with time**.
