# Supplementary figures and images for: Biofilm vs. Planktonic Lifestyle: Consequences for Pesticide 2,4-D Metabolism by Cupriavidus necator JMP134
Source: Front Microbiol. 2017 May 23;8:904. doi: 10.3389/fmicb.2017.00904 (PMC5440565; doi:10.3389/fmicb.2017.00904)

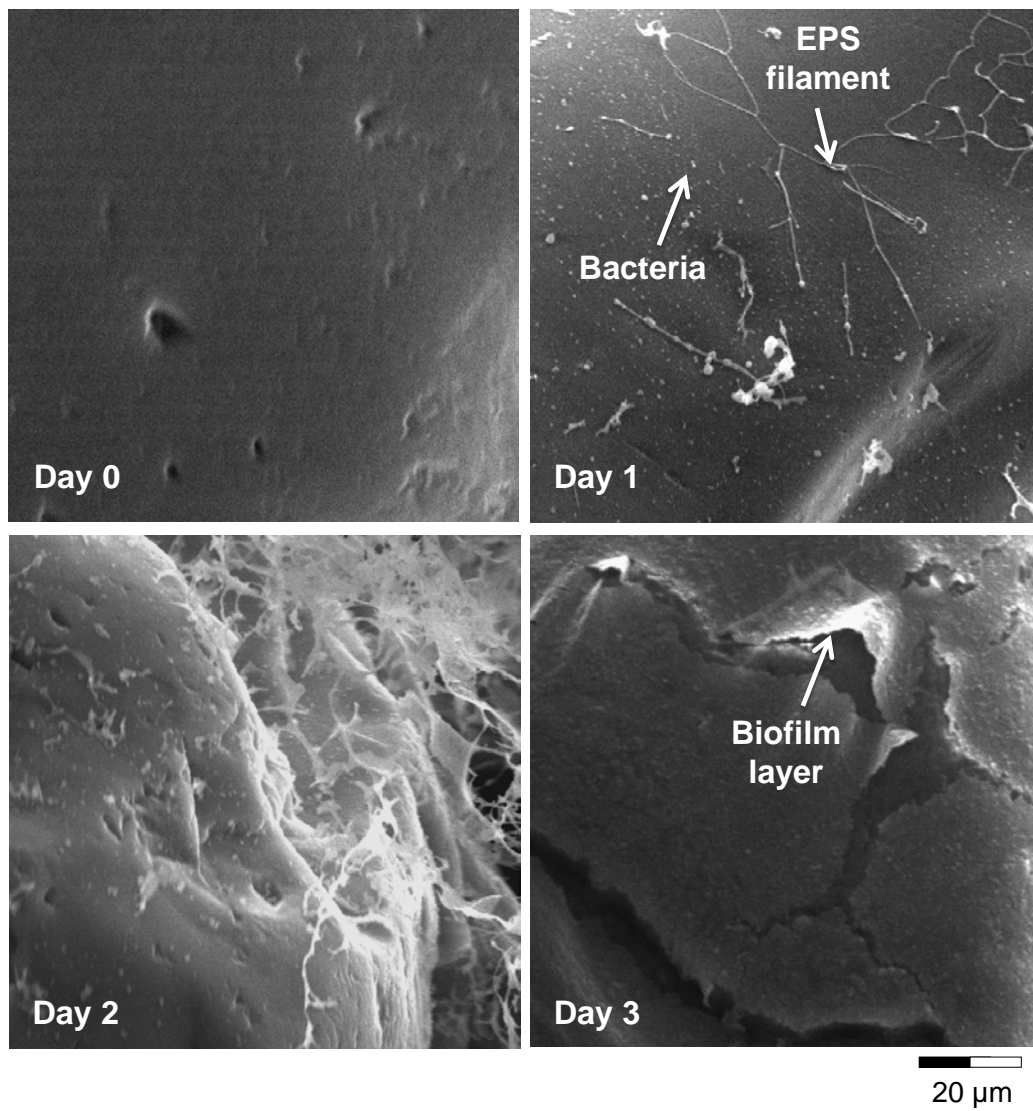

**Figure S1:** Scanning Electron Microscopy of the sand grains surface at 0, 1, 2 and 3 days of incubation.

Supplement: Figure S1 — Scanning Electron Microscopy of the sand grains surface at 0, 1, 2, and 3 days of incubation. [file Image1.pdf]
