# Supplementary material for: Biofilm vs. Planktonic Lifestyle: Consequences for Pesticide 2,4-D Metabolism by Cupriavidus necator JMP134
Source: Front Microbiol. 2017 May 23;8:904. doi: 10.3389/fmicb.2017.00904 (PMC5440565; doi:10.3389/fmicb.2017.00904)
Supplement: Figure S2 — Example of chromatograms (GC-FID) of the lipid fraction obtained from the sand samples at each sampling date. 2,4-D but no metabolites were detected. The other peaks corresponded to the bacterial fatty acids. [file Image2.pdf]

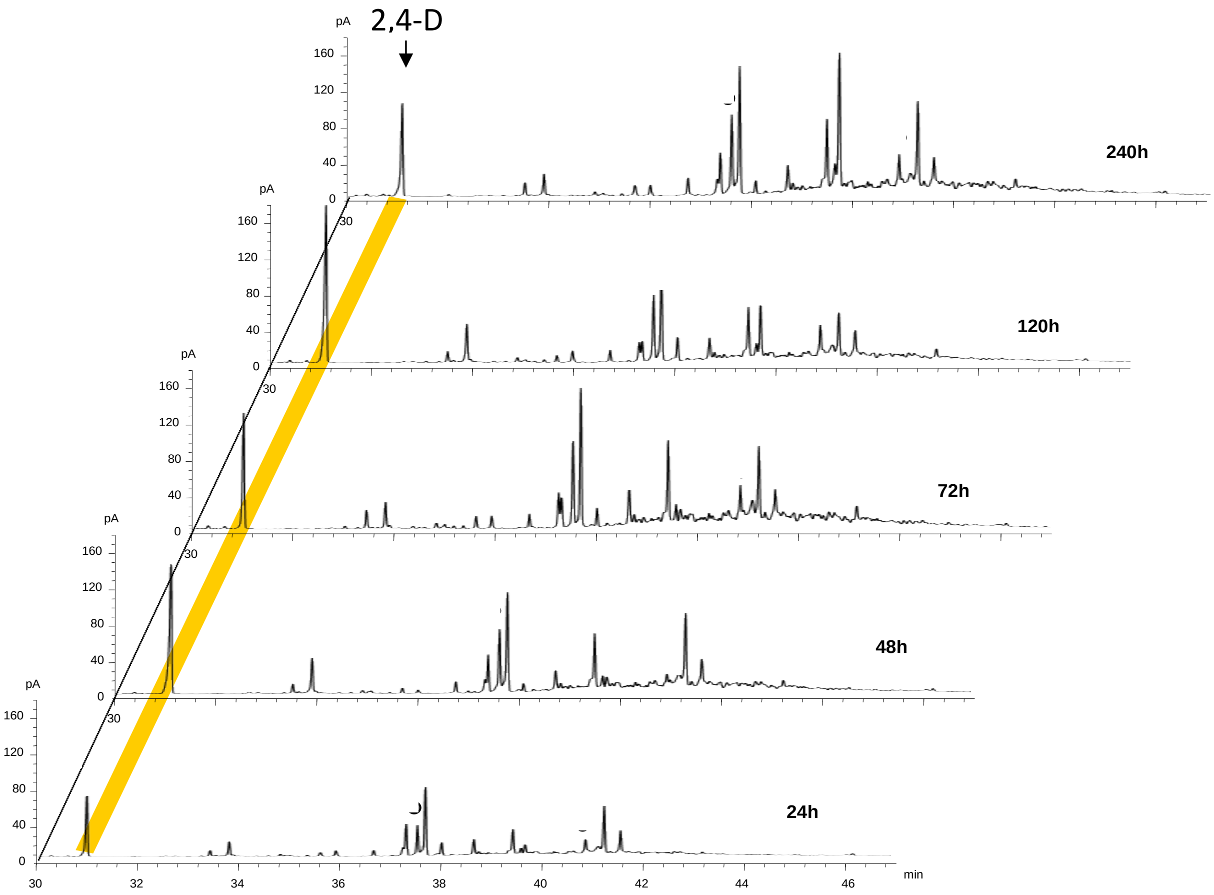

Figure S2: Example of chromatograms (GC-FID) of the lipid fraction obtained from the sand samples at each sampling date. 2,4-D but no metabolites were detected. The other peaks corresponded to the bacterial fatty acids.
