# Supplementary material for: Biofilm vs. Planktonic Lifestyle: Consequences for Pesticide 2,4-D Metabolism by Cupriavidus necator JMP134
Source: Front Microbiol. 2017 May 23;8:904. doi: 10.3389/fmicb.2017.00904 (PMC5440565; doi:10.3389/fmicb.2017.00904)
Supplement: Figure S3 — Composition (molar percentage) of C.necator JMP134 FAME profiles in the control (upper) and sand (lower) samples at each sampling date. Error bars correspond to the standard deviation calculated for 3 replicates. [file Image3.pdf]

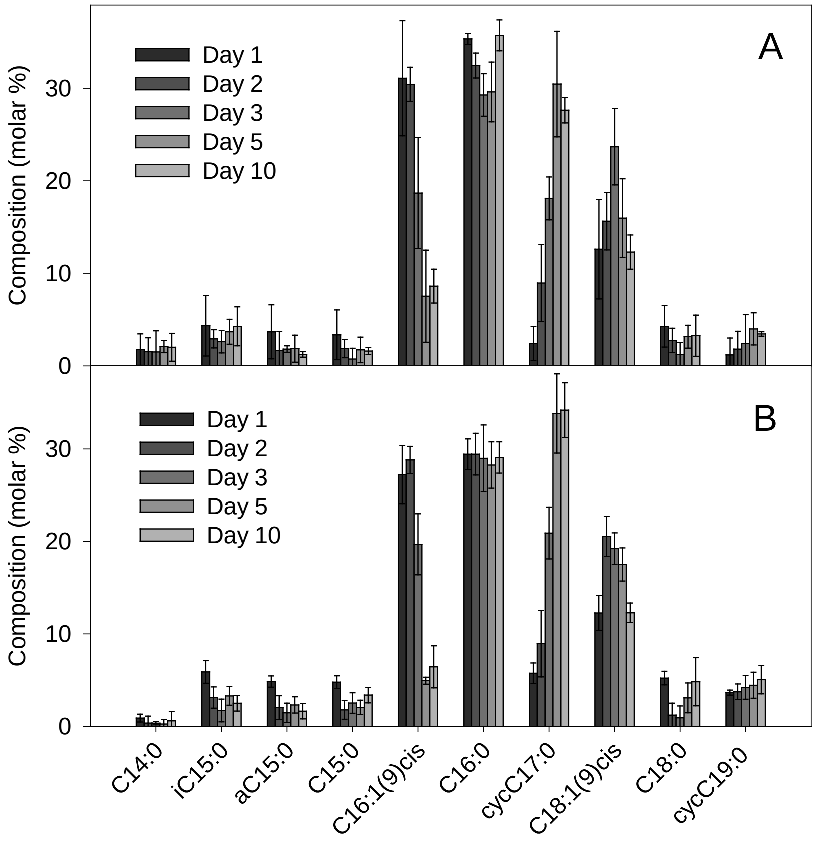


**Figure S3:** Composition (molar percentage) of *C.necator* JMP134 FAME profiles in the control (upper) and sand (lower) samples at each sampling date. Error bars correspond to the standard deviation calculated for 3 replicates.
