# Supplementary material for: Biofilm vs. Planktonic Lifestyle: Consequences for Pesticide 2,4-D Metabolism by Cupriavidus necator JMP134
Source: Front Microbiol. 2017 May 23;8:904. doi: 10.3389/fmicb.2017.00904 (PMC5440565; doi:10.3389/fmicb.2017.00904)
Supplement: Figure S4 — Proportion of CRing in the CO2 evolved during the 10 day incubation of C.necator JMP134 in the control (white circles) and sand (gray circles) microcosms. Error bars correspond to the standard deviation calculated for 3 replicates. Dash line represents the proportion of CRing in the substrate (2,4-D) used. [file Image4.pdf]

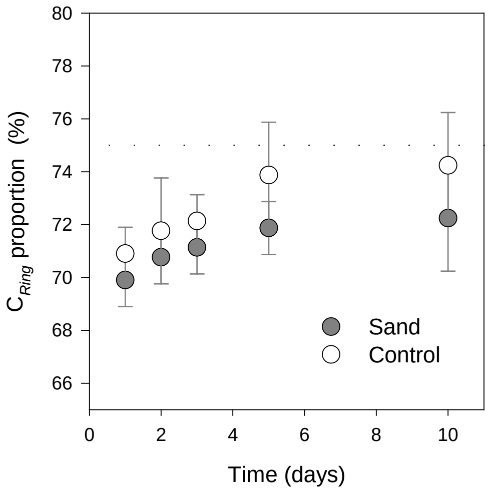

Figure S4: Proportion of C_{Ring} in the CO_2 evolved during the 10 day incubation of *C.necator* JMP134 in the control (white circles) and sand (grey circles) microcosms. Error bars correspond to the standard deviation calculated for 3 replicates. Dash line represents the proportion of C_{Ring} in the substrate (2,4-D) used.
