# Supplementary material for: Biofilm vs. Planktonic Lifestyle: Consequences for Pesticide 2,4-D Metabolism by Cupriavidus necator JMP134
Source: Front Microbiol. 2017 May 23;8:904. doi: 10.3389/fmicb.2017.00904 (PMC5440565; doi:10.3389/fmicb.2017.00904)
Supplement: Figure S5 — Proportion of CRing in each FAME of C.necator JMP134 for the control (A) and sand (B) microcosms at each sampling date. Error bars correspond to the standard deviation calculated for 3 replicates. The gray line represents the proportion of CRing in the substrate (2,4-D) used. [file Image5.pdf]

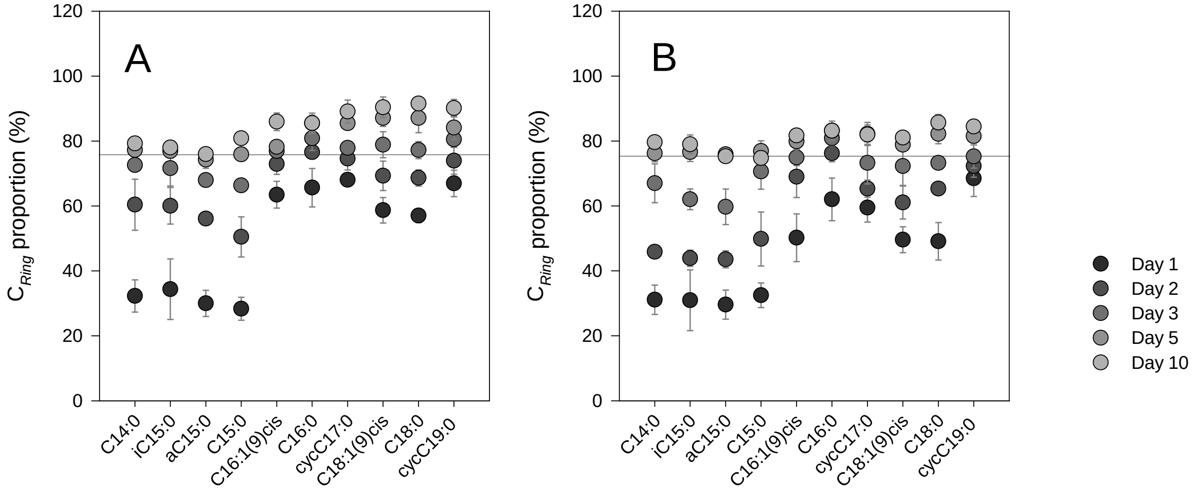

Figure S5: Proportion of C_{Ring} in each FAME of *C.necator* JMP134 for the control (A) and sand (B) microcosms at each sampling date. Error bars correspond to the standard deviation calculated for 3 replicates. The grey line represents the proportion of C_{Ring} in the substrate (2,4-D) used.
