# Supplementary material for: Biofilm vs. Planktonic Lifestyle: Consequences for Pesticide 2,4-D Metabolism by Cupriavidus necator JMP134
Source: Front Microbiol. 2017 May 23;8:904. doi: 10.3389/fmicb.2017.00904 (PMC5440565; doi:10.3389/fmicb.2017.00904)
Supplement: Table S1 — Evolution of the cyclopropyl/precursor ratio (cycC17:0 + cycC19:0/C16:1ω9c + C18:1ω9c) with time. [file Table1.pdf]

Table S1: Evolution of the cyclopropyl/precursor ratio (cycC17:0+cycC19:0/C16:1ω9c+C18:1ω9c) with time. Data are the means and standard deviations calculated for 3 replicates at each date of sampling.

Days	Control Samples		Samples with sand	
1	0,28	±0,02	0,24	±0,02
2	0,23	±0,02	0,26	±0,02
3	**0,48**	**±0,04**	**0,65**	**±0,05**
5	**1,47**	**±0,10**	**1,71**	**±0,11**
10	**1,49**	**±0,12**	**2,10**	**±0,17**
